# Protocol for the Optimune trial: a randomized controlled trial evaluating a novel Internet intervention for breast cancer survivors

**DOI:** 10.1186/s13063-019-3987-y

**Published:** 2020-01-29

**Authors:** Franziska Holtdirk, Anja Mehnert, Mario Weiss, Björn Meyer, Carsten Watzl

**Affiliations:** 1 Research Department, Gaia Group, Hans-Henny-Jahnn Weg 53, 22085 Hamburg, Germany; 2 Department of Medical Psychology and Medical Sociology, Section Psychosocial Oncology, University Hospital of Leipzig, Philipp-Rosenthal-Str. 55, 04103 Leipzig, Germany; 3 Technical University of Dortmund, Leibniz Research Centre (IfADo), Ardeystraße 67, 44139 Dortmund, Germany

**Keywords:** Breast cancer, Depression, Internet interventions

## Abstract

**Introduction:**

Depression and fatigue are common in breast cancer survivors, and their presence is associated with personal suffering and worse prognosis. While many women receive short-term psychological support in the acute treatment phase, this is rarely available in subsequent phases. Internet interventions for breast cancer survivors could provide additional psychological support, as they are easily accessible and may be effective. However, no trial has yet examined the effectiveness of an Internet intervention that provides cognitive behavioural therapy techniques plus lifestyle advice for this population. This trial aims to test whether *Optimune*, a novel Internet intervention we developed for that purpose, leads to improvements in quality of life and relevant lifestyle habits over the course of 3 to 6 months.

**Methods:**

This randomized controlled trial (RCT) will include 360 female breast cancer survivors who have completed the active tumour eradication phase. Participants will be recruited from various settings, including web-based advertisements and Internet forums in German-speaking countries. The main inclusion criteria are a breast cancer diagnosis less than 5 years ago and completion of acute treatment at least 1 month ago, as verified by discharge letter from an oncology treatment centre. Participants will be randomly assigned to either (1) a control group, in which they receive care as usual (CAU) and are given access to *Optimune* after a delay of 3 months (CAU/wait list control), or (2) a treatment group that may also use CAU and will receive 12-month access to *Optimune* immediately after randomization. The three primary endpoints are quality of life, physical activity and diet quality, assessed with the World Health Organization Quality of Life Questionnaire, the International Physical Activity Questionnaire and the Food Quality Questionnaire, at 3 months post-baseline; secondary outcomes include cancer-related fatigue, emotional stress, depression, anxiety, fear of progression, insomnia, usefulness of the programme and negative treatment effects. Online assessments are conducted at baseline (T0), 3 months (T1) and 6 months (T2).

**Discussion:**

Results of this RCT are expected to extend the body of knowledge with regard to the effectiveness of CBT-based Internet interventions for female breast cancer survivors.

**Trial registration:**

ClinicalTrials.gov, NCT03643640. Registered on 23 August 2018.

## Background

Approximately 70,000 women are diagnosed with breast cancer in Germany annually [1]. Breast cancer is the most frequent cancer among women worldwide, with an incidence of 2.09 million women each year [2]. The German guidelines for the treatment of breast cancer [3] delineate several acute treatment options, including surgery, radiation and aggressive combination chemotherapy. When these treatments are completed and patients are considered cancer-free, they are typically discharged from a phase of intense treatment to a phase of standardized monitoring for recurrence, with only a few specific therapies, such as treatment with antihormones, aromatase inhibitors or bisphosphonates.

While many women receive short-term psychological support in the phase of acute treatment, this is rarely the case in subsequent phases. Although women successfully treated for breast cancer often do not experience cancer recurrence during the monitoring phase, they frequently experience other symptoms. For example, an average of 27% of breast cancer survivors experience clinically significant fatigue following treatment [4, 5]. Furthermore, 40% of breast cancer survivors are affected by depression and 27% by anxiety, on average, and these symptoms typically persist for more than 5 years after diagnosis [6, 7]. There is also evidence that breast cancer survivors have higher levels of inflammation [8–10], which may contribute to their commonly elevated levels of depression, fatigue and anxiety [8, 9, 11].

Several pharmacological depression treatments have been shown to be effective among breast cancer survivors. A systematic review reported that the prescription of antidepressants is more common among breast cancer survivors (average rate of 23%) compared to other cancer types, even though some controversy persists as to whether selective serotonin re-uptake inhibitors interact with tamoxifen [12, 13]. Certain serotonin re-uptake inhibitors may alleviate depression, in part because of their effects on cytokines such as interleukin-6 (IL-6) and tumour necrosis factor (TNF)-α, whereas other antidepressants do not appear to reduce cytokine levels [14]. Of note, several studies showed improvement of depressive symptoms after administration of antiphlogistic agents, including nonsteroidal anti-inflammatory drugs (NSAIDs) and cytokine inhibitors [15, 16], suggesting a causative link between depressive symptoms and pro-inflammatory cytokines.

Certain psychological interventions for depression, including mindfulness-based stress reduction (MBSR), cognitive behavioural therapy (CBT) and supportive-expressive dynamic psychotherapy have also been shown to have an impact on inflammatory markers (e.g. IL-6, TNF-α) in several psychiatric and somatic diseases with comorbid depression [17–19]. A recent meta-analysis suggested that mindfulness meditation has an effect on inflammatory biomarkers such as C-reactive protein (CRP) or nuclear factor kappa-light-chain-enhancer of activated B cells (NF-ĸB) but seems neutral for a variety of other cytokines [20]. Moreover, a randomized controlled trial (RCT) with a sample of breast cancer survivors showed that yoga reduced fatigue and inflammation and showed a trend for the reduction of depressive symptoms [21]. Several other studies among cancer survivors have also shown that psychological interventions, including CBT and mindfulness-based treatment, may enhance quality of life as well as reduce pro-inflammatory signalling [22–24].

Even though CBT and perhaps other forms of psychotherapy are promising treatments for depression among breast cancer survivors, these psychological treatments are not always available [25]. Psycho-oncological services that offer such treatments are in short supply in Germany and elsewhere, particularly in rural areas [26]. Furthermore, various individual and systemic barriers may prevent breast cancer survivors from accessing psychological treatments, including stigma concerns, scepticism regarding psychotherapy, time constraints, disease-related restrictions, lack of motivation and perceived lack of necessity [27].

A potential barrier to pharmacological depression treatment is that physicians are often reluctant to prescribe antidepressants because of concerns over side effects or drug-drug interactions [12]. According to the German guidelines for psycho-oncology, psychotherapy as well as antidepressants are recommended for the treatment of depression in breast cancer survivors [28]. This recommendation is consistent with guidelines in several other countries, such as the USA and UK [29, 30]. To improve access and extend the range of available psychological treatment options for breast cancer survivors, Internet-based psychological interventions could play an important role [25, 31].

Given the potential utility of effective digital interventions, our team has developed a novel Internet-based intervention termed *Optimune*. This individually tailored Internet intervention targets female breast cancer survivors and was developed by Gaia, a German research-focused small-to-medium enterprise (SME) with more than 10 years of experience in the development of similar interventions. For example, 12 RCTs have demonstrated the efficacy of *Deprexis*, an Internet intervention for depression developed by the same team with the same technology [32–43]. A recent meta-analysis yielded an effect size of *g* = 0.54 for *Deprexis* [44]. Other evidence-based interventions developed by this group target anxiety disorders, fatigue in multiple sclerosis and harmful drinking as well as depression and anxiety in epilepsy [34, 45–47]. *Optimune* aims to reduce distress, depression, anxiety and, in particular, chronic inflammation in breast cancer survivors (see the Methods/design section for the intervention description).

The goal of this pragmatic RCT [48] is to test the effectiveness of this novel Internet intervention on improvements in quality of life and relevant lifestyle parameters (dietary and exercise habits) among breast cancer survivors over a period of 3 months, in comparison to a control group that only receives access to the intervention after 3 months. Both groups are free to utilize other treatments that may be required; thus, *Optimune* will be offered adjunctively to care as usual (CAU). Because control group participants can access *Optimune* after 3 months, they are on a wait list (WL) only with respect to the Internet intervention. Thus, the control group is best characterized as CAU/WL. It is hypothesized that, at 3 months post-baseline, participants randomized to the intervention group will report higher levels of quality of life, physical activity and diet quality compared to participants randomized to the CAU/WL control condition. In order to examine stability of intervention effects over time, data will also be collected at 6 months. Additional analyses will examine a broad range of secondary outcomes, including cancer-related fatigue, stress, depression, anxiety, fear of cancer progression and insomnia. The subjective usefulness of the intervention will also be examined. Exploratory analyses will be conducted to examine potential negative intervention effects.

## Methods/design

### Study design

**Fig. 1:**
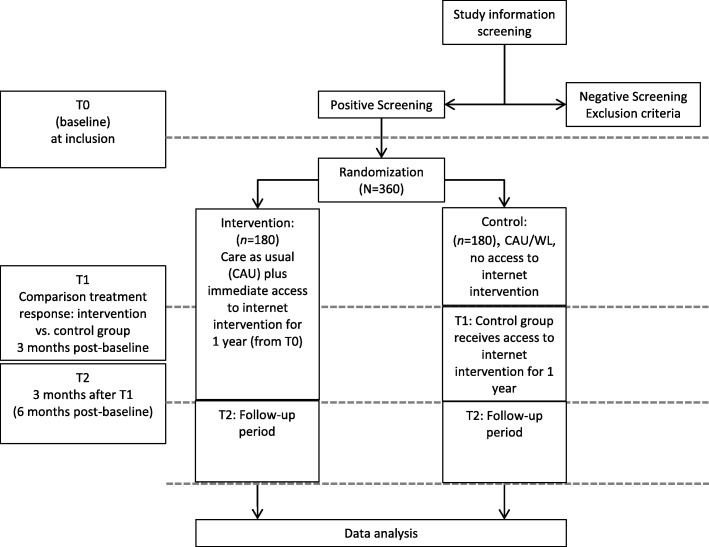
Flowchart of the study design

In this parallel-group RCT, participants will be randomized to two groups: (1) a control group, in which they may engage with any treatment and receive access to the Internet intervention after a delay of 3 months (CAU/WL), or (2) a treatment group that immediately receives access to the Internet intervention and may also use CAU (see Fig. 1). The trial aims to maximize external validity by examining the effect of adding a self-management intervention to the realities of current clinical care; it does not aim to maximize internal validity by selecting a maximally homogeneous patient group that does not reflect the clinical reality outside of trials [49]. Participants will be recruited consecutively from various sources, with every woman having an equal chance of being assigned to the intervention or control group (no block randomization). Given the pragmatic design, participants are not blinded to group assignment. Randomization will be performed by the Principal Investigator (PI), using a computer-generated sequence to generate the allocation sequence. However, the PI will not have any access to any participant data prior to performing the randomization. The other researchers who do have access to participant data in order to clarify inclusion/exclusion criteria will not have access to the randomization sequence. Thus, concealed allocation will be ensured.

The content and design of this RCT are in accordance with the guidelines for clinical trial protocols as specified by the Standard Protocol Items: Recommendations for Interventional Trials (SPIRIT) 2013 statement [50]. The SPIRIT checklist is provided as Additional file 2. Moreover, this RCT is registered at ClinicalTrials.gov (NCT03643640), and any changes to this protocol will be described in this trial registry. The trial will conform to methodological standards recommended by the Consolidated Standards of Reporting Trials (CONSORT) statement [51].

### Recruitment and assessment

Study participants will be recruited via different channels. First, the study will be advertised online, and interested participants will be informed via Google ads, Internet forums and a dedicated Facebook page. Furthermore, treatment centres will be informed about the goals and procedures of the study and provided with informational material such as flyers and posters. Other recruitment channels will include patient associations, support groups and health insurance companies. Women who are interested in participating will be referred to the study homepage [52]. Internet users who use search engines such as Google with certain search terms (e.g. breast cancer self-help, breast cancer study, *Optimune*, etc.) will be shown a short ad that refers to the study homepage; potential participants may also be referred to the study homepage via other means (e.g. a recommendation by their treating oncologist/gynaecologist or their health insurance company). On the study homepage, interested participants will be invited to sign up with their name and e-mail address. A member of the research team will then contact potential participants by e-mail in order to invite them to the online baseline assessment (T0). Prior to completing the online baseline measures, further study information will be presented and online informed consent will be obtained.

After providing consent and completing the T0 online assessment, eligible participants will be asked to verify their diagnosis and therapies by providing the research team with a discharge letter from their oncology treatment centre or clinic. After receipt of this and confirmation of all inclusion/exclusion criteria, eligible participants will be randomized. One member of the study team will monitor registration for the programme and contact the participants if they do not sign up within 1 week after access to the intervention has been granted. Participants will be contacted again after 3 months (T1) and 6 months (T2) to complete the respective online assessments (see Table 1), and they will be reminded up to two times if they do not respond to the initial invitations. Previous studies evaluating similar interventions have shown that effects on psychosocial outcomes can be observed after a relatively brief period of 8 to 12 weeks [34, 36, 40–43, 46]. To evaluate the stability of effects and to allow the examination of potential effects beyond the 3 months time point, online assessments will also be conducted for both groups 6 months post-randomization.

**Table 1:**
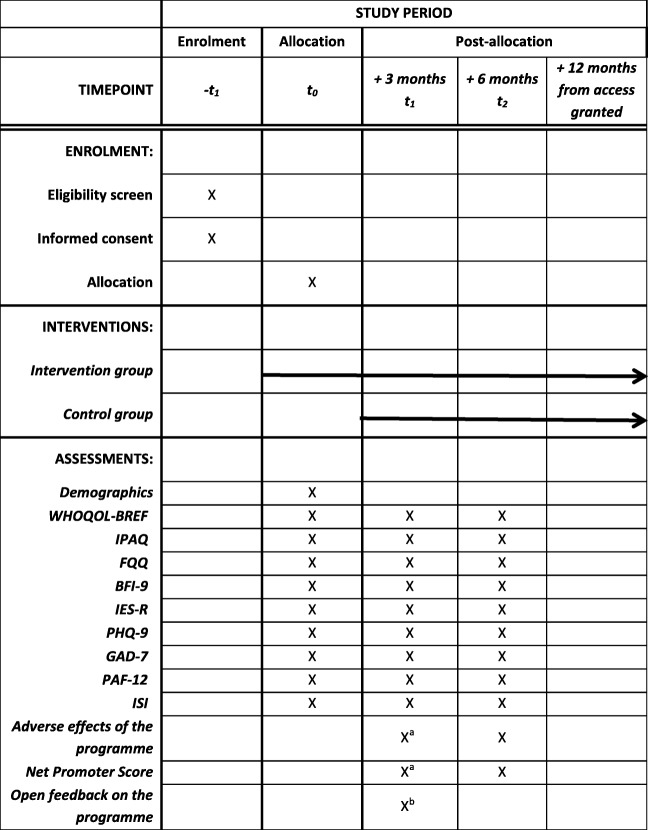
Study interventions, measures and measurement points

*WHOQOL-BREF* World Health Organization Quality of Life Questionnaire, *IPAQ* International Physical Activity Questionnaire, *FQQ* Food Quality Questionnaire, *BFI-9* Brief Fatigue Inventory, *IES-R* Impact of Event Scale-Revised, *PHQ-9* Patient Health Questionnaire, 9 items, *GAD-7* Generalized Anxiety Disorder 7-item questionnaire, *PAF-12* Fear of Progression Questionnaire, *ISI* Insomnia Severity Index

^a^Only for intervention group

^b^For each group: 15 days after access was granted

### Inclusion criteria

Women will be included if they have received a breast cancer diagnosis less than 5 years ago and acute treatment for breast cancer (such as surgery, chemotherapy or radiation treatment) has been completed at least 1 month prior to study entry. Prophylactic treatment with antihormone therapy such as tamoxifen, aromatase inhibitors or bisphosphonates is permitted. Eligible participants must be between 30 and 70 years of age; provide informed consent; be competent in the German language; and be willing to provide the discharge letter to verify their diagnoses and therapies.

### Intervention

*Optimune* is an Internet intervention for female breast cancer survivors. The intervention was developed by a multidisciplinary team of clinical psychologists, CBT therapists, physicians, software engineers, graphic artists and professional speakers, among others, who are affiliated with Gaia in Hamburg, Germany. Beyond established CBT techniques targeting depression, anxiety and fatigue, this intervention engages users in therapeutic techniques that have been shown to have beneficial effects on immune system functioning and inflammation, including sleep and stress management (e.g. mindfulness-based techniques) and lifestyle change (dietary and physical activity advice). The dimensions addressed in the programme are consistent with the German treatment guidelines for breast cancer survivors [28, 53].

The development team strove to include only techniques that are supported by evidence, and relevant citations are provided throughout the programme, accompanied by brief summaries of the respective journal article or book (annotated bibliography). Overall, 240 citations are provided across all *Optimune* modules. Content is continuously automatically adapted via software algorithms in order to match users’ individual preferences, concerns and needs. Content is conveyed in the format of interactive ‘simulated dialogues’ in which users read information or listen to recordings and then select one of several pre-programmed response options. The intervention uses a responsive web-design approach and can be accessed on a password-protected, secure (https-encrypted) website via any computer or smartphone. Illustrations, audio recordings and optional daily text messages with motivational content accompany the programme (see Additional file 1 for screenshots).

Throughout the development process, breast cancer survivors were continuously involved to test preliminary versions and provide feedback to ensure the quality of the software, consistent with development guidelines [54]. Expert feedback was sought from several physicians specializing in the treatment of patients with breast cancer, and the literature on CBT for breast cancer survivors was reviewed [24, 55, 56]. Like other software programs developed by this group, the intervention was produced on a proprietary software (broca®) developed by Gaia. The software-based intervention uses cloud computing with fast global access and is securely hosted in an ISO-27001-certified data centre located in Germany.

The *Optimune* intervention can be accessed over a period of 1 year. Depending on factors such as reading speed, preference for greater or lesser depth and desire to listen to audio recordings, each module can be completed in approximately 30–45 min. It would be possible to work through the entire intervention in as little as approximately 6 h. As in other interventions developed by our group [34, 39, 40], there is no fixed or generic sequence in which modules must be completed, nor is there a minimum number of sessions that must be finished. Users are invited to freely explore the intervention and let themselves be guided within each section by the algorithm-driven sequences that the intervention generates. Users are also informed that they can discontinue the intervention at any time if they feel that it is not helpful or may even be harmful.

The *Optimune* intervention comprises 16 modules that cover four broad domains: (1) psychological well-being, (2) dietary coaching, (3) exercise and physical activity coaching and (4) sleep management. An introductory module provides the user with an overview of the purpose, functions and time frame of the intervention. Participants are engaged in interactive sequences in which the role of inflammation and the immune system in breast cancer are briefly explained. The introductory module further addresses topics such as current emotional well-being (including possible symptoms of stress, depression and anxiety) as well as exercise and dietary habits. Participants are provided with a summary (’personal profile’) of their responses, and they receive initial personalized recommendations. Furthermore, participants are encouraged to complete each new module once it is activated for them, and to allow sufficient time between sessions to work on the tasks and exercises introduced in each session. They are informed that intervention access is provided for a 12-month period. The four intervention domains are outlined in the following subsections.

#### Psychological well-being

This domain provides an interactive overview of the role positive and negative emotions may play in inflammation and immune system functioning, and it introduces individually tailored exercises to reduce stress and improve well-being. Mindfulness and mental imagery exercises (including audio recordings) are introduced and practiced, and concepts/techniques such as activity scheduling, behavioural activation and basic psychological need satisfaction are introduced. Similar to the modules in the other domains (other than the introductory module), every module in this domain begins with a brief review of tasks that were assigned in the previous module.

#### Dietary coaching

Participants are provided with a brief overview of nutrition and dietary habits in the context of inflammation. The technique of stimulus control is introduced, and participants are encouraged to reduce the availability of potentially pro-inflammatory foods (e.g. heavily processed foods, crisps, pizza, doughnuts, cake, red meat, lemonade) from the household and restock with ’anti-inflammatory foods’ (e.g. primarily certain vegetables and fruits, such as blueberry, raspberry, broccoli, spinach, avocado, olive oil, turmeric). As in all other modules, evidence-based behaviour-change techniques such as goal setting, action planning and mental contrasting (of pros and cons or goal obstacles and solutions) are used.

#### Exercise/physical activity coaching

The first module within this domain starts with a personalized exploration of current exercise and physical activity habits, followed by an interactive review of relevant research findings and their implications for daily life. Participants are encouraged to set realistic and attainable exercise goals for the coming weeks and use prompts/reminders to facilitate the adoption of new exercise habits. Mental imagery exercises and other behaviour-change techniques are used (e.g. case examples to encourage modelling, identifying barriers and problem solving, agreeing on behavioural contracts, reviewing if-then plans, action-planning and rehearsing implementation intentions and using positive mental imagery and self-motivational statements).

#### Sleep management

This domain begins with an interactive exploration of current sleep quality and potential difficulties (insomnia symptoms and impairment in quality of life). Psychoeducational content regarding the importance of sleep for immune system functioning is provided, followed by an interactive exploration of evidence-based CBT techniques for overcoming insomnia (sleep hygiene, establishing regular sleep-wake rhythms, etc.). A personalized plan with suggestions for healthy sleep habits is provided, and users are encouraged to apply the presented techniques in order to optimize their sleep quality in the coming weeks.

### Outcome measurements

All outcome measures will be used in their German version and assessed via a secure, encrypted online survey service. Participants will be invited via e-mail to complete the online self-report questionnaires. If they do not respond to the initial invitation, a member of the research team will send them up to two reminder e-mails.

### Primary outcomes

Three equally important primary endpoints are defined: quality of life, dietary quality and physical exercise. Differences in change from baseline between study groups at 90 days from randomization will be assessed with the World Health Organization Quality of Life Questionnaire (WHOQOL-BREF), the International Physical Activity Questionnaire (IPAQ) and the Food Quality Questionnaire (FQQ). Success of the trial is defined as demonstrating a significant intervention effect on at least one of these endpoints. Given that multiple outcomes increase the familywise error rate, Bonferroni’s adjustment will be used [57].

Quality of life will be measured using the World Health Organization’s *WHOQOL-BREF*, which is a shorter version of the original instrument. The WHOQOL-BREF [58, 59] is a 26-item, 5-point rating scale with good to excellent psychometric properties (reliability and validity [60]). It measures the following domains: physical health, psychological health, social relationships and environment. Response options range from 1, indicating ’very dissatisfied’, to 5, indicating ’very satisfied’ and relate to the last 2 weeks.

Physical activity will be measured using the short version of the *International Physical Activity Questionnaire (IPAQ-SF)*. The short version of the IPAQ is a 7-item questionnaire that assesses the types of intensity of people’s physical activity and sitting time performed during their daily lives. Open-ended questions surrounding individuals’ last 7-day recall of physical activity are considered to estimate total physical activity (minutes/week) and time spent sitting. The IPAQ-SF has been evaluated as an acceptable measure to assess activity or fitness, as shown in a systematic review [61]. Overall, its reliability is acceptable to good.

Diet quality will be measured using the *FQQ*. The FQQ is a newly developed short food frequency questionnaire which includes 10 food items on a 5-point rating scale. It focuses on fruit, vegetables, high-sugar foods, processed food, meat and drinks. The aim of the tool is to measure diet quality. Response options range from 0, indicating ’daily’, to 3, indicating ’once or never’. A global diet quality score can be obtained by averaging all the items on the FQQ. This questionnaire is based on other, similar questionnaires, and its psychometric properties are being evaluated for the first time in this study.

### Secondary outcomes

The following secondary outcomes will be assessed using self-report. Each instrument will be administered at T0, T1 and T2, except for the *Net Promoter Score (NPS)* and the items regarding negative effects of the programme.

- Cancer-related fatigue using the *Brief Fatigue Inventory (BFI-9)* [62, 63]
- Cancer-related emotional stress using the standardized *Impact of Event Scale-Revised (IES-R)* [64]
- Depression using the standardized 9-item *Patient Health Questionnaire (PHQ-9 )* [65, 66]
- Anxiety using the standardized questionnaire *Generalized Anxiety Disorder 7 item (GAD-7)* [67, 68]
- Fear of progression using the standardized *PA-F12* (Progredienzangstfragebogen [Fear of Progression]) questionnaire [69, 70]
- Insomnia using the standardized *Insomnia Severity Index (ISI)* [71]
- Subjective usefulness of the programme using the *NPS* [72] and several items to assess negative effects of the programme.

Additionally, participants will be asked about specific illness-related parameters including recurrence of breast cancer (local relapse or remote metastasis), frequency of common cold or virus flu, frequency of unscheduled medical encounters, body mass index and the parallel use of psychotherapy at T0, T1 and T2. Furthermore, participants will be asked for open feedback on the programme 15 days after access has been granted.

### Sample size calculation and analysis

To estimate the target sample size, an a priori power analysis was performed with g*power (Version 3.1.9.2) [73]. In order to detect a small to medium effect (*d* = 0.35), a total sample size of 346 participants will be required to achieve a power of 0.80 with an adjusted alpha level of 0.0167 (Bonferroni adjustment because of the three primary endpoints; 0.05/3). An effect of *d* = 0.35 has been shown in previous reviews on psychosocial outcomes in patients with cancer [74–76]. Therefore, we aim to enrol 180 participants per group (total target *N* = 360). Because missing data will be replaced by multiple imputation, the target sample size is not adjusted for anticipated attrition.

In line with recommendations of the CONSORT eHealth statement, we will conduct both intention-to-treat (ITT) and per-protocol (PP) analyses using appropriate statistical procedures such as analysis of covariance (ANCOVA) for primary and secondary outcome measures [51, 77]. Missing data will be imputed for ITT and PP analyses at the primary endpoints after 3 months with multiple imputation (100 imputations, 50 iterations) based on sociodemographic data and available outcomes, following current methodological recommendations [78]. ITT analyses will include data from all randomized participants, with missing data replaced by multiple imputation. For PP analyses, in line with previous studies [40, 45], we will include data from control group participants plus data from intervention group participants who have used the intervention for at least 60 min on at least four separate occasions (i.e. four sessions). For PP analyses, missing data will also be replaced using multiple imputation, in line with recommendations [79]. Bonferroni-adjusted ANCOVAs will be conducted for each primary outcome to test for between-group effects after 3 months using baseline measures as covariates, as recommended for trials with pre- and post-measurement [80].

The reasons that lead to the exclusion of participants will be reported and analysed for patterns [81], that is, systematic differences in their occurrence between the control group and intervention group.

## Discussion

The present study aims to make a contribution to the literature on Internet interventions for breast cancer survivors, and to test the effectiveness of a potential new treatment option that may help improve the quality of care for this group. There is a need for innovative interventions in this area, given that depression and fatigue are exceedingly common in women after the acute treatment phase, and psycho-oncological treatment provision is often insufficient, particularly in rural areas. To our knowledge, *Optimune* is the first Internet intervention for breast cancer survivors that uses an extensive tailoring approach to convey CBT content to users. It has been specifically designed to address the needs of breast cancer survivors and uses a responsive web-design approach to maximize personal relevance and ensure easy usability.

An advantage of this trial is that diagnoses will be verified by a clinician, which is rare in studies of Internet interventions. Additional methodological strengths include follow-up assessments and the inclusion of an array of validated measures for the primary and secondary outcomes. Of note, potential negative treatment effects will also be assessed, which is rarely done but required to establish the safety of novel Internet interventions. Another strength is that participants will be asked to provide open feedback about the programme.

Several limitations must also be acknowledged. First, the trial does not include an active control condition, but comparisons with CAU are regarded as common and appropriate features of pragmatic RCTs. Second, because we could not identify a brief and previously validated food questionnaire that adequately covers the food groups targeted by our intervention, we included a new questionnaire that was developed for this study. We regard this study as a first step towards examining this questionnaire’s psychometric properties, but we acknowledge the limitation that further research will be required to establish its reliability and validity. Third, the target sample size may yield insufficient statistical power for the detection of small effects, especially if attrition rates are high. As recommended by the CONSORT statement, we plan to replace missing data by appropriate procedures (e.g. multiple imputation), and we expect that the sample size will allow us to detect effects of medium magnitude, which could be considered clinically relevant. Due to resource limitations, recruitment of a large sample is not possible in this trial. Fourth, the inclusion of three primary outcome variables is unusual. However, given that the intervention aims to improve quality of life as well as change dietary and exercise habits, we regarded all three as equally important outcomes, each of which deserves equal consideration. To reduce the familywise error rate, the Bonferroni correction will be applied. The fifth limitation concerns the lack of clinician outcome ratings. Self-report questionnaires will be used, which are efficient and validated but also carry the risk of introducing biases such as social desirability. An additional limitation is that the developers of the intervention that is being tested are involved in this trial. We note, however, that this is not unusual and that several steps are taken to ensure the scientific integrity of data collection and analysis. To avoid potential developer bias, data monitoring will be performed by the Principal Investigator (CW), who is an established independent researcher in immunology with no financial ties to the developers or other relevant conflicts of interest. The trial has been registered, and results will be published regardless of study outcome.

In conclusion, the present study will test the effectiveness of the new self-management intervention *Optimune,* which will be offered as an adjunct to care as usual. If the results from this trial demonstrate the effectiveness of the *Optimune* programme, this would suggest that this Internet intervention could serve as a useful treatment tool with the potential to enhance the overall quality of care for breast cancer survivors.

### Trial status

The protocol version number is OPT 01, issued 13 November 2019. Participant recruitment began on 1 September 2018, and was completed on 1 October 2019. Collection of follow-up data will continue until 1 April 2020.

## Supplementary information

**Additional file 1. **Exemplary screenshots of the *Optimune* programme, showing exemplary content of the four domains covered in *Optimune*.

**Additional file 2.** SPIRIT 2013 checklist: recommended items to address in a clinical trial protocol and related documents.

## Data Availability

Not applicable.

## Abbreviations

BFI-9: Brief Fatigue Inventory
CAU: Care as usual
CBT: Cognitive behavioural therapy
FQQ: Food Quality Questionnaire
GAD-7: Generalized Anxiety Disorder 7-item
IES-R: Impact of Event Scale-Revised
IPAQ short: Short version of the International Physical Activity Questionnaire
ISI: Insomnia Severity Index
ITT: Intention-to-treat
MBSR: Mindfulness-based stress reduction
PA-F12: Progredienzangstfragebogen (Fear of Progression Questionnaire)
PHQ-9: Patient Health Questionnaire, 9 items
PI: Principal Investigator
PP: Per-protocol
RCT: Randomized controlled trial
WHOQOL-BREF: World Health Organization Quality of Life Questionnaire
WL: Wait list

## References

[1] Robert Koch-Institut. Brustkrebs (Mammakarzinom). 2017. https://www.krebsdaten.de/Krebs/DE/Content/Krebsarten/Brustkrebs/brustkrebs_node.html. Accessed 12 Mar 2019.

[2] Bray F, Ferlay J, Soerjomataram I, Siegel RL, Torre LA, Jemal A (2018). Global cancer statistics 2018: GLOBOCAN estimates of incidence and mortality worldwide for 36 cancers in 185 countries. CA Cancer J Clin.

[3] Kreienberg R, Albert U, Follmann M, Kopp I, Kühn T, Wöckel A (2013). Interdisziplinäre S3-Leitlinie für die Diagnostik, Therapie und Nachsorge des Mammakarzinoms. Senologie - Zeitschrift für Mammadiagnostik und -therapie.

[4] Abrahams HJG, Gielissen MFM, Schmits IC, Verhagen CAHHVM, Rovers MM, Knoop H (2016). Risk factors, prevalence, and course of severe fatigue after breast cancer treatment: a meta-analysis involving 12 327 breast cancer survivors. Ann Oncol.

[5] Bower JE, Ganz PA, Desmond KA, Bernaards C, Rowland JH, Meyerowitz BE (2006). Fatigue in long-term breast carcinoma survivors: a longitudinal investigation. Cancer..

[6] Maass SWMC, Roorda C, Berendsen AJ, Verhaak PFM, de Bock GH (2015). The prevalence of long-term symptoms of depression and anxiety after breast cancer treatment: a systematic review. Maturitas..

[7] Von Ah D, Kang D-H (2008). Correlates of mood disturbance in women with breast cancer: patterns over time. J Adv Nurs.

[8] Bower JE, Ganz PA, Aziz N, Fahey JL (2002). Fatigue and proinflammatory cytokine activity in breast cancer survivors. Psychosom Med.

[9] Collado-Hidalgo A, Bower JE, Ganz PA, Cole SW, Irwin MR (2006). Inflammatory biomarkers for persistent fatigue in breast cancer survivors. Clin Cancer Res.

[10] Pierce BL, Ballard-Barbash R, Bernstein L, Baumgartner RN, Neuhouser ML, Wener MH (2009). Elevated biomarkers of inflammation are associated with reduced survival among breast cancer patients. J Clin Oncol.

[11] Dowlati Y, Herrmann N, Swardfager W, Liu H, Sham L, Reim EK (2010). A meta-analysis of cytokines in major depression. Biol Psychiatry.

[12] Sanjida S, Janda M, Kissane D, Shaw J, Pearson S-A, DiSipio T (2016). A systematic review and meta-analysis of prescribing practices of antidepressants in cancer patients. Psychooncology..

[13] Juurlink D (2016). Revisiting the drug interaction between tamoxifen and SSRI antidepressants. BMJ..

[14] Hannestad J, DellaGioia N, Bloch M (2011). The effect of antidepressant medication treatment on serum levels of inflammatory cytokines: a meta-analysis. Neuropsychopharmacology..

[15] Köhler O, Benros ME, Nordentoft M, Farkouh ME, Iyengar RL, Mors O (2014). Effect of anti-inflammatory treatment on depression, depressive symptoms, and adverse effects: a systematic review and meta-analysis of randomized clinical trials. JAMA Psychiatry..

[16] Raison CL, Rutherford RE, Woolwine BJ, Shuo C, Schettler P, Drake DF (2013). A randomized controlled trial of the tumor necrosis factor antagonist infliximab for treatment-resistant depression: the role of baseline inflammatory biomarkers. JAMA Psychiatry.

[17] Del Grande da Silva G, Wiener CD, Barbosa LP, Gonçalves Araujo JM, Molina ML, San Martin P (2016). Pro-inflammatory cytokines and psychotherapy in depression: results from a randomized clinical trial. J Psychiatr Res.

[18] Moreira FP, de Azevedo Cardoso T, Mondin TC, de Mattos Souza LD, Silva R, Jansen K (2015). The effect of proinflammatory cytokines in Cognitive Behavioral Therapy. J Neuroimmunol.

[19] Walsh E, Eisenlohr-Moul T, Baer R (2016). Brief mindfulness training reduces salivary IL-6 and TNF-α in young women with depressive symptomatology. J Consult Clin Psychol.

[20] Black DS, Slavich GM (2016). Mindfulness meditation and the immune system: a systematic review of randomized controlled trials. Ann N Y Acad Sci.

[21] Kiecolt-Glaser JK, Bennett JM, Andridge R, Peng J, Shapiro CL, Malarkey WB (2014). Yoga’s impact on inflammation, mood, and fatigue in breast cancer survivors: a randomized controlled trial. J Clin Oncol.

[22] Bower JE, Crosswell AD, Stanton AL, Crespi CM, Winston D, Arevalo J (2015). Mindfulness meditation for younger breast cancer survivors: a randomized controlled trial. Cancer..

[23] Carlson LE, Speca M, Patel KD, Goodey E (2003). Mindfulness-based stress reduction in relation to quality of life, mood, symptoms of stress, and immune parameters in breast and prostate cancer outpatients. Psychosomatic Medicine.

[24] McGregor BA, Antoni MH (2009). Psychological intervention and health outcomes among women treated for breast cancer: a review of stress pathways and biological mediators. Brain Behav Immun.

[25] Cuijpers P (2015). Psychotherapies for adult depression: recent developments. Current Opinion in Psychiatry.

[26] Zimmermann-Schlegel V, Hartmann M, Sklenarova H, Herzog W, Haun MW (2017). Accessibility, availability, and potential benefits of psycho-oncology services: the perspective of community-based physicians providing cancer survivorship care. Oncologist..

[27] Mohr DC, Ho J, Duffecy J, Baron KG, Lehman KA, Jin L (2010). Perceived barriers to psychological treatments and their relationship to depression. J Clin Psychol.

[28] Leitlinienprogramm Onkologie. Psychoonkologische Diagnostik, Beratung und Behandlung von erwachsenen Krebspatienten. 2014. https://www.leitlinienprogramm-onkologie.de/fileadmin/user_upload/Downloads/Leitlinien/Psychoonkologieleitlinie_1.1/LL_PSO_Langversion_1.1.pdf. Accessed 7 Mar 2019.

[29] Runowicz CD, Leach CR, Henry NL, Henry KS, Mackey HT, Cowens-Alvarado RL (2016). American Cancer Society/American Society of Clinical Oncology Breast Cancer Survivorship Care Guideline. CA Cancer J Clin.

[30] National Institute for Clinical Excellence (2002). Improving outcomes in breast cancer: manual update.

[31] Kazdin AE, Blase SL (2011). Rebooting psychotherapy research and practice to reduce the burden of mental illness. Perspect Psychol Sci.

[32] Beevers CG, Pearson R, Hoffman JS, Foulser AA, Shumake J, Meyer B (2017). Effectiveness of an internet intervention (Deprexis) for depression in a United States adult sample: a parallel-group pragmatic randomized controlled trial. J Consult Clin Psychol.

[33] Berger T, Hämmerli K, Gubser N, Andersson G, Caspar F (2011). Internet-based treatment of depression: a randomized controlled trial comparing guided with unguided self-help. Cogn Behav Ther.

[34] Berger T, Urech A, Krieger T, Stolz T, Schulz A, Vincent A (2017). Effects of a transdiagnostic unguided Internet intervention ('velibra’) for anxiety disorders in primary care: results of a randomized controlled trial. Psychol Med.

[35] Fischer A, Schröder J, Vettorazzi E, Wolf OT, Pöttgen J, Lau S (2015). An online programme to reduce depression in patients with multiple sclerosis: a randomised controlled trial. Lancet Psychiatry.

[36] Fuhr K, Fahse B, Hautzinger M, Gulewitsch M (2018). Erste Erfahrungen zur Implementierbarkeit einer internet-basierten Selbsthilfe zur Überbrückung der Wartezeit auf eine ambulante Psychotherapie. PPmP - Psychother Psych Med Psych.

[37] Gräfe V, Greiner W (2017). Internet based treatment of depressive symptoms — a health economic evaluation of costs and benefits. Value Health.

[38] Klein JP, Berger T, Schröder J, Späth C, Meyer B, Caspar F (2016). Effects of a psychological internet intervention in the treatment of mild to moderate depressive symptoms: results of the evident study, a randomized controlled trial. Psychother Psychosom.

[39] Meyer B, Berger T, Caspar F, Beevers CG, Andersson G, Weiss M (2009). Effectiveness of a novel integrative online treatment for depression (Deprexis): randomized controlled trial. J Med Internet Res.

[40] Meyer B, Bierbrodt J, Schröder J, Berger T, Beevers CG, Weiss M (2015). Effects of an Internet intervention (Deprexis) on severe depression symptoms: randomized controlled trial. Internet Interv.

[41] Moritz S, Schilling L, Hauschildt M, Schröder J, Treszl A (2012). A randomized controlled trial of internet-based therapy in depression. Behav Res Ther.

[42] Schröder J, Brückner K, Fischer A, Lindenau M, Köther U, Vettorazzi E (2014). Efficacy of a psychological online intervention for depression in people with epilepsy: a randomized controlled trial. Epilepsia..

[43] Zwerenz R, Becker J, Knickenberg RJ, Siepmann M, Hagen K, Beutel ME (2017). Online self-help as an add-on to inpatient psychotherapy: efficacy of a new blended treatment approach. Psychother Psychosom.

[44] Twomey C, O’Reilly G, Meyer B (2017). Effectiveness of an individually-tailored computerised CBT programme (Deprexis) for depression: a meta-analysis. Psychiatry Res.

[45] Meyer Björn, Weiss Mario, Holtkamp Martin, Arnold Stephan, Brückner Katja, Schröder Johanna, Scheibe Franziska, Nestoriuc Yvonne (2019). Effects of an epilepsy‐specific Internet intervention (Emyna) on depression: Results of the ENCODE randomized controlled trial. Epilepsia.

[46] Pöttgen J, Moss-Morris R, Wendebourg J-M, Feddersen L, Lau S, Köpke S (2018). Randomised controlled trial of a self-guided online fatigue intervention in multiple sclerosis. J Neurol Neurosurg Psychiatry.

[47] Zill JM, Christalle E, Meyer B, Härter M, Dirmaier J. The effectiveness of an internet intervention aimed at reducing alcohol consumption in adults. Deutsches Aerzteblatt Online. 2019. 10.3238/arztebl.2019.0127. 10.3238/arztebl.2019.0127 PMC6454801 30940341

[48] Hotopf M (2002). The pragmatic randomised controlled trial. Adv Psychiatr Treat.

[49] Zwarenstein M, Treweek S (2009). What kind of randomised trials do patients and clinicians need?. Evid Based Med.

[50] Chan A-W, Tetzlaff JM, Altman DG, Laupacis A, Gøtzsche PC, Krleža-Jerić K (2013). SPIRIT 2013 statement: defining standard protocol items for clinical trials. Ann Intern Med.

[51] Eysenbach G, CONSORT-EHEALTH Group (2011). CONSORT-EHEALTH: Improving and Standardizing Evaluation Reports of Web-based and Mobile Health Interventions. J Med Internet Res.

[52] Optimune study web site. Hamburg: Gaia Group; 2018. https://optimune-studie.de.

[53] Kuemmel S, Schmidt M. AGO Guidelines Breast Version 2016.1D: Komplementäre Therapien Hormontherapie „Survivorship“ (Rezidiv-Prävention). 2016. https://www.ago-online.de/fileadmin/downloads/leitlinien/mamma/Maerz2016/de/2016D%2024_Komplementaere%20Therapie.pdf. Accessed 20 May 2019.

[54] Yardley L, Morrison L, Bradbury K, Muller I (2015). The person-based approach to intervention development: application to digital health-related behavior change interventions. J Med Internet Res.

[55] Antoni MH (2003). Stress management intervention for women with breast cancer.

[56] Kvillemo P, Bränström R (2014). Coping with breast cancer: a meta-analysis. PLOS One.

[57] Vickerstaff V, Ambler G, King M, Nazareth I, Omar RZ (2015). Are multiple primary outcomes analysed appropriately in randomised controlled trials? A review. Contemp Clin Trials.

[58] The WHOQOL Group (1998). Development of the World Health Organization WHOQOL-BREF Quality of Life Assessment. Psychol Med.

[59] World Health Organization. WHOQOL-BREF : introduction, administration, scoring and generic version of the assessment : field trial version, December 1996. Geneva: WHO; 1996. https://apps.who.int/iris/handle/10665/63529?show=full. Accessed 12 Feb 2019.

[60] Skevington SM, Lotfy M, O’Connell KA (2004). The World Health Organization’s WHOQOL-BREF quality of life assessment: psychometric properties and results of the international field trial. A Report from the WHOQOL Group. Qual Life Res.

[61] Lee PH, Macfarlane DJ, Lam T, Stewart SM (2011). Validity of the International Physical Activity Questionnaire Short Form (IPAQ-SF): a systematic review. Int J Behav Nutr Phys Act.

[62] Mendoza TR, Wang XS, Cleeland CS, Morrissey M, Johnson BA, Wendt JK (1999). The rapid assessment of fatigue severity in cancer patients. Cancer..

[63] Radbruch L, Sabatowski R, Elsner F, Everts J, Mendoza T, Cleeland C (2003). Validation of the German version of the Brief Fatigue Inventory. J Pain Symptom Manag.

[64] Creamer M, Bell R, Failla S (2003). Psychometric properties of the Impact of Event Scale-Revised. Behav Res Ther.

[65] Kroenke K, Spitzer RL (2002). The PHQ-9: a new depression diagnostic and severity measure. Psychiatr Ann.

[66] Kroenke K, Spitzer RL, Williams JBW (2001). The PHQ-9: validity of a brief depression severity measure. J Gen Intern Med.

[67] Löwe B, Decker O, Müller S, Brähler E, Schellberg D, Herzog W (2008). Validation and standardization of the Generalized Anxiety Disorder Screener (GAD-7) in the general population. Med Care.

[68] Spitzer RL, Kroenke K, Williams JBW, Löwe B (2006). A brief measure for assessing generalized anxiety disorder: the GAD-7. Arch Intern Med.

[69] Herschbach P, Berg P, Engst-Hastreiter U, Waadt S, Duran G, Henrich G. Entwicklung und Evaluation eines Therapieprogramms zur Bewältigung von Progredienzangst. Munich: Klinik und Poliklinik für Psychosomatische Medizin und Psychotherapie, Klinikum rechts der Isar, Technische Universität München; 2006.

[70] Herschbach P, Dankert A, Duran-Atzinger G, Waadt S, Engst-Hastreiter U, Keller M (2001). Diagnostik von Progredienzangst – Entwicklung eines Fragebogens zur Erfassung von Progredienzangst bei Patienten mit Krebserkrankungen, Diabetes mellitus und entzündlich-rheumatischen Erkrankungen in der Rehabilitation.

[71] Morin CM, Belleville G, Bélanger L, Ivers H (2011). The Insomnia Severity Index: psychometric indicators to detect insomnia cases and evaluate treatment response. Sleep..

[72] Keiningham TL, Aksoy L, Cooil B, Andreassen TW, Williams L (2008). A holistic examination of Net Promoter. J Database Mark Cust Strategy Manag.

[73] Cohen J (1992). A power primer. Psychol Bull.

[74] Ledesma D, Kumano H (2009). Mindfulness-based stress reduction and cancer: a meta-analysis. Psycho-Oncology..

[75] Musial F, Büssing A, Heusser P, Choi K-E, Ostermann T (2011). Mindfulness-based stress reduction for integrative cancer care — a summary of evidence. Forschende Komplementärmedizin/Research in Complementary Medicine.

[76] Tatrow K, Montgomery GH (2006). Cognitive behavioral therapy techniques for distress and pain in breast cancer patients: a meta-analysis. J Behav Med.

[77] Gupta S (2011). Intention-to-treat concept: a review. Perspectives Clin Res.

[78] Li P, Stuart EA, Allison DB (2015). Multiple imputation: a flexible tool for handling missing data. JAMA..

[79] Jakobsen JC, Gluud C, Wetterslev J, Winkel P (2017). When and how should multiple imputation be used for handling missing data in randomised clinical trials — a practical guide with flowcharts. BMC Med Res Methodol.

[80] O’Connell NS, Dai L, Jiang Y, Speiser JL, Ward R, Wei W (2017). Methods for analysis of pre-post data in clinical research: a comparison of five common methods. J Biom Biostat.

[81] EMEA (1998). ICH E9 statistical principles for clinical trials.

