# Supplementary material for: Protocol for the Optimune trial: a randomized controlled trial evaluating a novel Internet intervention for breast cancer survivors
Source: Trials. 2020 Jan 29;21:117. doi: 10.1186/s13063-019-3987-y (PMC6990478; doi:10.1186/s13063-019-3987-y)
Supplement: Supplementary file 1 — Additional file 1. Exemplary screenshots of the Optimune programme, showing exemplary content of the four domains covered in Optimune. [file 13063_2019_3987_MOESM1_ESM.docx]

**
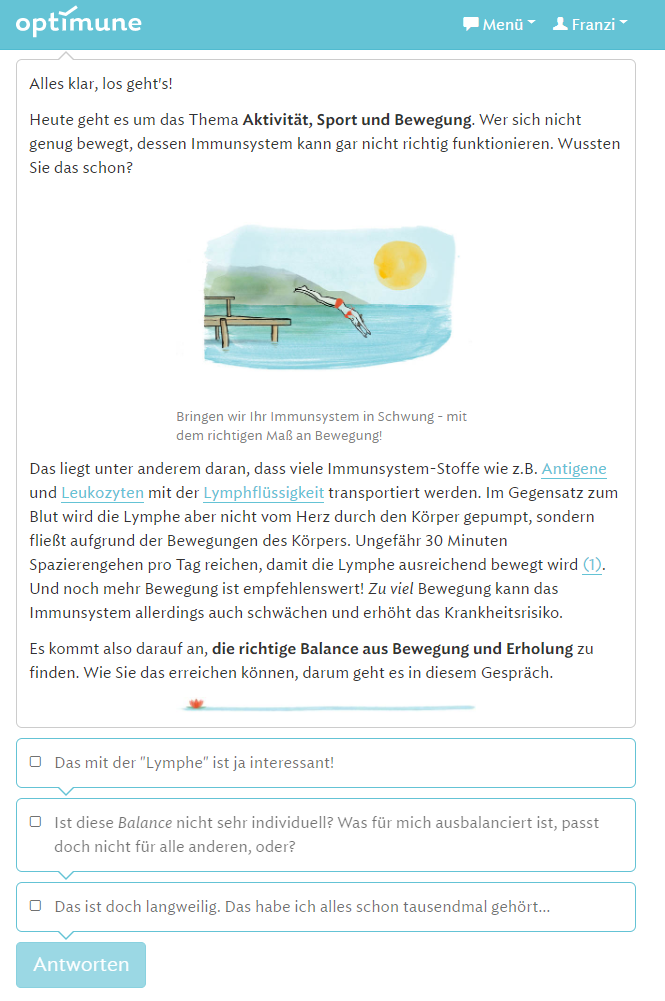
**

Optimune – example physical activity coaching

**
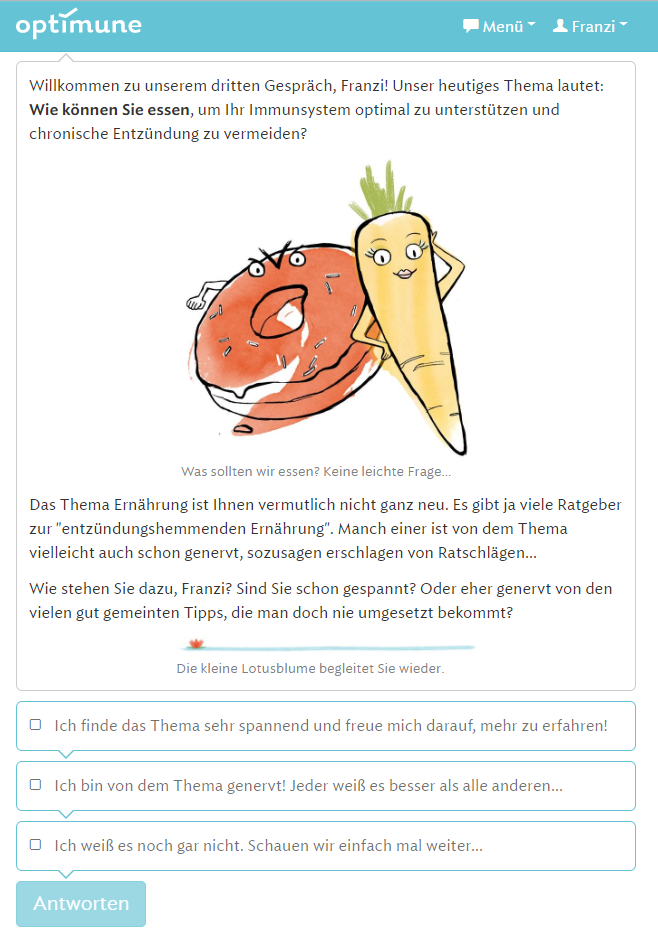
**

Optimune – example dietary coaching

**
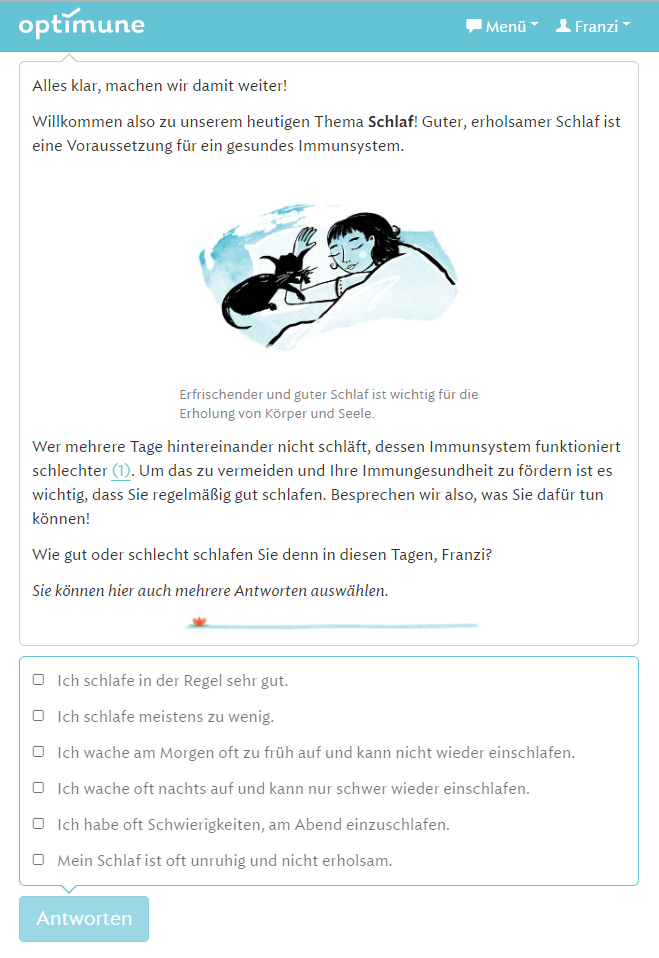
**

Optimune – example sleep management

**
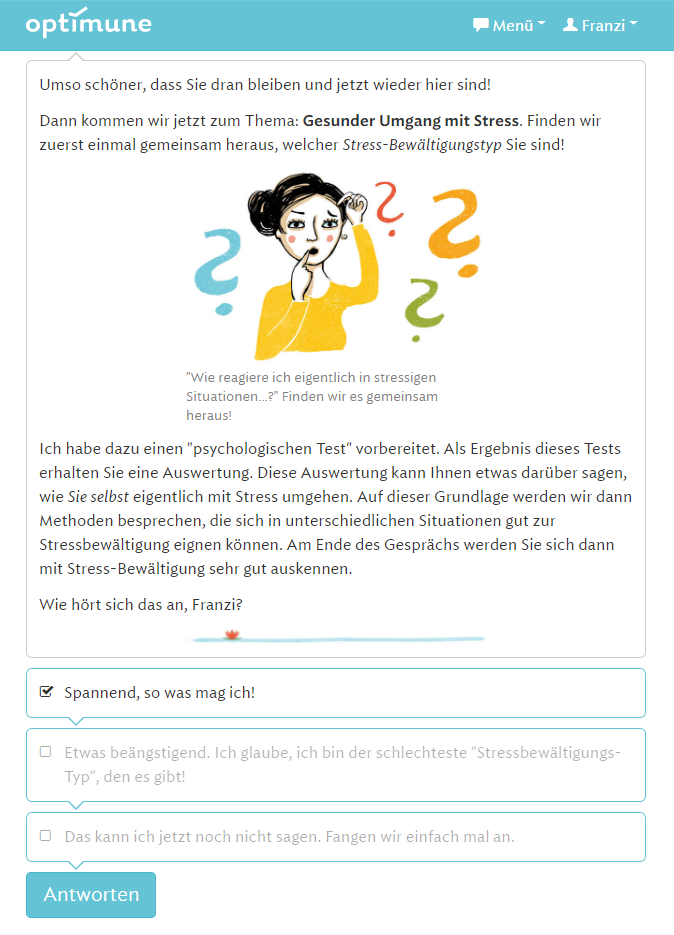
**

Optimune – example psychological well-being
